# Response to the Letter to the Editor: “Immunosuppressive therapy management during sepsis in kidney transplant recipients: a prospective multicenter study”

**DOI:** 10.1186/s13613-025-01599-w

**Published:** 2025-10-27

**Authors:** Valentin Rivet, Lara Zafrani

**Affiliations:** 1 Medical Intensive Care Unit, Saint-Louis Hospital, Assistance Publique- Hôpitaux de Paris, Paris, France; 2 INSERM UMR 1342, Université Paris Cité, Paris, France

Dear Editor,

We thank you for giving us the opportunity to respond to the letter to the editor previously published [1] concerning our article [2].

The correspondents raise an important issue concerning the heterogeneity of infection types, particularly the predominance of SARS-CoV-2 pneumonia and suggest stratified analyses. As shown in Tables 6 and 7, we performed subgroup analyses. In patients with SARS-CoV-2 pneumonia, discontinuation of at least one immunosuppressive therapy (IST) or calcineurin inhibitor (CNI) was not significantly associated with ICU mortality, acute kidney injury (AKI), major adverse kidney events (MAKE) at ICU discharge, or MAKE 180. In patients with septic shock, only mycophenolic acid (MPA) discontinuation was significantly associated with MAKE 180 in univariate analysis. To account for potential confounding from COVID-19-specific treatments, we included SARS-CoV-2 status and its interaction with MPA discontinuation in our model. Results were consistent across COVID-19 and non-COVID-19 patients (Table S6).

They also point out that focusing on a single drug may not reflect the complexity of clinical practice, as kidney transplant recipients usually receive multidrug regimens. We agree that overall immunosuppressive burden is key. In our cohort, MPA discontinuation was the most frequent and clinically relevant adjustment, which is why we examined it specifically. Given the heterogeneity of IST management and limited sample size, distinct patterns could not be reliably analyzed. We addressed this limitation in our discussion. A standardized tool to quantify net immunosuppression would indeed facilitate individualized management but is not yet available.

Finally, the correspondents note that the low incidence of acute rejection in our study may underestimate the true risk, without systematic surveillance (protocol biopsies, DSA, immune profiling). We agree that standardized monitoring approaches would provide a more comprehensive assessment. As follow-up was performed by different transplant centers with variable protocols, standardized surveillance was not feasible. We concur that closer collaboration with nephrology teams and standardized follow-up strategies would strengthen future studies.

We thank the correspondents for their thoughtful comments, which have allowed us to further clarify our findings and highlight avenues for future research.

## Data Availability

The datasets used and/or analyzed during the current study are available from the corresponding author on reasonable request.
